# Polydopamine Linking Substrate for AMPs: Characterisation and Stability on Ti6Al4V

**DOI:** 10.3390/ma13173714

**Published:** 2020-08-22

**Authors:** Zuzanna Trzcińska, Marc Bruggeman, Hanieh Ijakipour, Nikolas J. Hodges, James Bowen, Artemis Stamboulis

**Affiliations:** 1 School of Metallurgy and Materials, University of Birmingham, Edgbaston, Birmingham B15 2TT, UK; zutrzcinska@outlook.com (Z.T.); MXB1082@student.bham.ac.uk (M.B.); HXI756@student.bham.ac.uk (H.I.); 2 School of Biosciences, University of Birmingham, Edgbaston, Birmingham B15 2TT, UK; n.hodges@bham.ac.uk; 3 School of Chemical Engineering, University of Birmingham, Edgbaston, Birmingham B15 2TT, UK; james.bowen@open.ac.uk

**Keywords:** Ti6Al4V, polydopamine, antimicrobial peptides, cathelicidin, KR-12

## Abstract

Infections are common complications in joint replacement surgeries. Eradicated infections can lead to implant failure. In this paper, analogues of the peptide KR-12 derived from the human cathelicidin LL-37 were designed, synthesised, and characterised. The designed antimicrobial peptides (AMPs) were attached to the surface of a titanium alloy, Ti6Al4V, by conjugation to a polydopamine linking substrate. The topography of the polydopamine coating was evaluated by electron microscopy and coating thickness measurements were performed with ellipsometry and Atomic Force Microscopy (AFM). The subsequently attached peptide stability was investigated with release profile studies in simulated body fluid, using both fluorescence imaging and High-Performance Liquid Chromatography (HPLC). Finally, the hydrophobicity of the coating was characterised by water contact angle measurements. The designed AMPs were shown to provide long-term bonding to the polydopamine-coated Ti6Al4V surfaces.

## 1. Introduction

Infection are the most common complications of joint replacement surgery, with nosocomial or hospital-acquired infections ranking as the sixth leading cause of death, presenting a major healthcare challenge [1]. Infection can lead to extended inflammation at the site of the surgery, thus causing the rejection and failure of the implant [2]. Although the administration of antibiotics significantly reduces the risk of postsurgical infections, bacterial biofilm production on the implant surface or untimely administration of antibiotics will reduce their effectiveness [3]. 

The use of titanium in dental and orthopaedic implants is well established due to titanium’s strength, stiffness, and corrosion resistance. Titanium also shows seamless integration with the surrounding tissues due to its excellent biocompatibility [4,5]. The drawback of using titanium implants is their susceptibility to bacterial colonisation on the surfaces of the implants [6]. To combat the formation of biofilms on implant surfaces, the time-controlled release of various antibiotic coatings has previously been investigated [7,8]. However, the release of the antibiotics below the level of the minimum inhibitory concentration (MIC) is known to produce antibiotic-resistant strains of bacteria. Higher levels of antibiotic release have been shown to be toxic to the surrounding tissues. The increase of antibiotic-resistant bacteria has led to the search for an alternative method of antimicrobial protection [9].

Antimicrobial peptides, which are a part of the innate immune system of all living organisms, have broad-spectrum activity against many microorganisms, such as Gram-positive bacteria, Gram-negative bacteria, viruses, and fungi [10,11,12,13]. Moreover, they can inhibit biofilm formation and induce its dissolution, as well as attract phagocytes to further induce natural defence mechanisms [14]. The mechanisms of action of antimicrobial peptides (AMPs) against bacteria are not fully understood due to the high diversity of these peptides. Nevertheless, it is widely accepted that bacterial cell death is due to the interaction of cationic AMPs with negatively charged phospholipids on the bacterial membrane, which lead to the loss of membrane structural integrity, and eventually cell death [10,11,12,13]. AMPs exhibit a strong preference for specific membrane compositions, allowing them to be selective towards bacterial cell membranes, but not mammalian or plant cells [14]. Currently, only a few AMPs are used clinically due to limiting factors, such as the high cost of peptide synthesis, their susceptibility to proteolytic degradation, and their unknown long-term toxicology profiles [1,14,15]. Here, analogues of the peptide KR-12 derived from the human cathelicidin LL-37 were designed due to its established antimicrobial activity and lack of mammalian cell toxicity, as originally found by Jacob et al. [16].

To introduce the antimicrobial peptides stably on a surface, different types of coatings can be employed. A popular approach is the use of polydopamine (pDA), a strong adhesive mussel-inspired polymer, due to its low cost, simplicity of application, and improved biocompatibility [17,18,19]. Similarly to mussel adhesive proteins, the adhesive properties of the pDA are owed to quinine and catechol groups, which create chelating structures with metals. Additionally, after polymerisation, pDA can be further functionalised with amine-containing nucleophiles, such as proteins and peptides. This allows the application of a pDA coating to Ti6Al4V, where the in-house designed analogues of the peptide KR-12 are subsequently covalently bonded to the pDA coating. 

## 2. Materials and Methods

### 2.1. Peptide Design

The shortest active peptide fragment derived from cathelicidin LL-37, KR12, was used as a template to design three peptides of varying antimicrobial activity. The peptide sequences and various properties are summarised in Table 1.

### 2.2. Peptide Synthesis, Purification 

Peptides dyed with carboxyfluorescein (5(6)-FAM) were synthesised in-house following conventional solid-phase peptide synthesis (SPPS). After synthesis, the peptides were purified to show a minimum purity of >95%. Peptides without the dye exhibited a purity of >98% and were purchased from ProteoGenix, Schiltigheim, France. All the amino acids used in SPPS (i.e., Fmoc–Ala–OH, Fmoc–Asp(OtBu)–OH, Fmoc–Glu(OtBu)–OH, Fmoc–Phe–OH, Fmoc–Gly–OH, Fmoc–Ile–OH, Fmoc–Lys(Boc)–OH, Fmoc–Leu–OH, Fmoc–Asn–OH, Fmoc–Gln–OH, Fmoc–Pro–OH, Fmoc–Arg(Pbf)–OH) were purchased from AGTC Bioproducts Ltd., Itlings Lane Hessle, UK, with a purity of >98 % purity, which were side-chain protected where appropriate. Preloaded Wang resin with Fmoc-protected amino acids (Fmoc–Arg(Pbf) Wang, Fmoc–Glu(OtBu) Wang, Fmoc–Lys(Boc)–OH) with peptide substitutions in the range of 0.21–5.3 mmol/g and o-benzotriazole–N,N,N′,N′–tetramethyl–uronium–hexafluoro-phosphate (HBTU) of >98% purity were purchased from NovaBiochem, Merck Life Science UK Limited, Watford, UK. Anhydrous N,N dimethylformide (DMF), anhydrous dichloromethane (DCM), 5(6)-FAM, piperidine/DMF 20% mix (*v*/*v*), ninhydrin, trifluoroacetic acid (TFA), and triisopropylsilane (TIPS) were all HPLC grade with >99% purity, which were purchased from Sigma Aldrich, Merck Life Science UK Limited, Dorset, UK. Anhydrous diethyl ether of HPLC grade used for precipitation of peptides was purchased from Fisher Scientific UK Ltd., Loughborough, UK. SPPS reactions were carried out in Aldrich^®^ System 45™ vessels with caps and fritted discs purchased from Sigma Aldrich, Merck Life Science UK Limited, Dorset, UK. Acetonitrile and TFA used for peptide purification and for purity analysis with high-pressure liquid chromatography (HPLC) were of HPLC grade and were purchased from Sigma Aldrich, Merck Life Science UK Limited, Dorset, UK. 

Once ready for cleavage, the dried Wang resin beads were weighed and transferred from the reaction vessel into a 50 mL round-bottom flask equipped with a magnetic stirrer. A fresh cleavage mixture made of TFA, dH_2_O, and TIPS (*v*/*v*/*v* = 95/2.5/2.5) was then prepared and 10 mL per 0.1 g of dried resin beads was added to the Wang resin beads in the round-bottom flask. The flask was then carefully closed and wrapped with aluminium foil to prevent photo-bleaching. The reagents were left to react under gentle and continuous stirring for 3–5 h. Subsequently, the Wang resin beads were removed by vacuum filtration and the peptides were dissolved in the cleavage mixture. The peptides were then precipitated by adding the solution dropwise to ice-cold diethyl ether. To ensure maximum precipitation, the diethyl ether was kept overnight at −20 °C. The precipitate was then separated from the supernatant by centrifugation at 13,500 rpm and air-drying overnight at −20 °C. The dried crude peptide powder was dissolved in dH_2_O and then freeze-dried using a LyoDry Midi freeze dryer (MechaTech Systems Ltd., Bristol, UK).

Labelling of the synthesised peptides with the fluorescence dye 5(6)-FAM was performed while the molecules were still attached to the Wang resin. The reaction is similar to the amino acid coupling. A mixture of 5 equiv 5(6)-FAM and 4.9 equiv HBTU dissolved in DMF (≈3 mL) was placed in a reaction vessel containing a known amount of washed Wang resin with a previously deprotected N-terminal peptide sequence. The reaction vessel was wrapped with aluminium foil to prevent photo-bleaching and was placed on an orbital shaker. The reaction mixture was left to react overnight under continuous shaking (≈200 rpm). It was then washed three times with DMF (6 mL) and three times with DCM (6 mL). After the washing procedure, the resin beads were dried under vacuum until all of the DCM was removed.

### 2.3. Peptide Characterisation

#### 2.3.1. High-Pressure Liquid Chromatography 

All peptides were purified with preparative HPLC using a Thermo Scientific Dionex Summit preparative HPLC system (Thermo Fisher Scientific Ltd., Loughborough, UK). Their purity was tested with a Thermo Scientific Dionex Summit analytical HPLC system. Both systems were equipped with C18 columns, water with 0.05% TFA was used as the hydrophilic medium, and acetonitrile with 0.05% TFA was used as the hydrophobic medium. The detection of molecules was conducted by measuring the UV absorbance of the amide bond at a wavelength of 210 nm. For preparative HPLC, a total of 2 mL of crude peptide dissolved in a mixture of acetonitrile and water (*v*/*v* = 1) was injected into the C18 column, while for analytical HPLC 100 µL of a similar peptide solution was added to the C18 column. In both cases, the columns were run for 60 min with a linear gradient of solvents starting at 100% dH_2_O and 0% acetonitrile, finishing at 0% water and 100% acetonitrile. 

#### 2.3.2. Mass Spectrometry 

After purification, acetonitrile was evaporated with the use of a rotary evaporator and the peptides were recovered by freeze-drying. Subsequently, the peptides were analysed on a Waters Micromass LCT TOF spectrometer (Waters UK, Wilmslow, UK) using electrospray ionisation in the School of Chemistry Mass Spectrometry facility. Prior to mass analysis, the peptide samples were dissolved in water. After the purification of the crude peptides, a purity of ≥95% was achieved and the theoretical mass agreed with the calculated mass displayed in Table 2.

#### 2.3.3. Minimum Inhibitory Concentration (MIC) Values of Peptides

The minimum inhibition concentration (MIC) is used to determine the lowest concentration of antimicrobial agent needed to inhibit the visible growth of a bacteria strain after overnight incubation. All the bacteria strains were kindly provided by Dr Mark Webber of the Quadram Institute Bioscience (Norwich, UK; *Escherichia coli* (*E. coli*, I364), *Pseudomonas aeruginosa* (*P. aeruginosa*, PAO1) and *Staphylococcus aureus* (*S. aureus,* F77/NCTC8532)). Lysogen broth (LB broth) and agar were purchased from Sigma-Aldrich, Merck Life Science UK Limited, Dorset, UK. Fresh LB agar culture plates were prepared by pouring ≈10 mL of an autoclaved warm mixture of LB broth (2.5%) and bacteriological agar (1.5%) dissolved in dH_2_O. The agar plate was streaked and incubated overnight at 36 °C. A single bacterial colony was chosen and grown in 5 mL broth overnight under agitation at 36 °C. Subsequently, 50 μL of LB broth was added to wells 2–12 of a 96-well culture plate. AMPs were diluted to a concentration of 256 μg/mL, added to well 1, and diluted two-fold down to column 11. Column 12 was left empty, with no AMPs added. Then, 50 μL of the diluted overnight bacteria culture was added to the wells and incubated at 36 °C for 18 h to allow bacteria to grow. After incubation, the well plates were examined for bacterial growth and the lowest concentration of AMPs where clear liquid was observed was assumed to be the minimum inhibitory concentration. Three measurements for each peptide and against each type of bacteria were performed and the average values were obtained. 

### 2.4. Sample Preparation

Titanium alloy grade 5 (Ti6Al4V) plates with dimensions of 15 cm × 15 cm and a thickness of 0.1 cm were purchased from William Gregor Ltd., London, UK, and cut into 1 cm × 1 cm plates. Then, the plates were mounted in conducting Bakelite and polished to mirror finish. Three steps were used during polishing of the plates. First, Bakelite-mounted Ti6Al4V plates were ground with MD-Piano of 220 grit, with water used as a lubricant. Then, a DiaDuo-2 diamond of 9 μm grain size suspended in water and an MD-Largo polishing plate were used. Finally, a colloidal suspension (OP-S) activated with ammonia solution was used and polished on MD-Chem polishing disc. All the polishing materials and equipment were purchased from Struers Ltd., Rotherham, UK. After polishing the plates to a mirror finish, the highly polished surfaces were secured with electrical tape to prevent the introduction of scratches and Bakelite was broken down to release the mounted Ti6Al4V plates. The tape was then removed from the plates and any impurities introduced on the Ti6Al4V surfaces during the previous steps were removed by cleaning the plates in an ultrasonic bath with water (15 min) and acetone (15 min). The plates were dried overnight in a desiccator and were used within 48 h after cleaning.

Having been cleaned and polished to a mirror finish, Ti6Al4V plates were placed inside a 24-well cell culture plate with the polished side facing upwards. Dopamine, purchased from Sigma-Aldrich, was dissolved to a final concentration of 5 mg/mL in 50 mM Tris buffer (Fisher Scientific UK Ltd., Loughborough, UK) at pH = 8.5. Then, 1.5 mL of dopamine solution was transferred into the cell culture plates containing the Ti6Al4V plates. Subsequently, the prepared plates were placed in the dark for 24 h without a cover to allow simultaneous dopamine polymerisation in air and metallic surface coating. Ti6Al4V plates coated with polydopamine (pDA) were washed to remove any loose pDA particles and placed in a new set of 24-well cell culture plates. The AMP solution was then prepared by dissolving peptides in a concentration equal to the MIC value for each peptide in 50 mM Tris buffer at pH 7.4. Then, 1.5 mL of this solution was transferred into the cell culture plates with the pDA-coated Ti6Al4V plates. The cell culture plates were then kept in the dark without a cover for 24 h to allow conjugation of the peptides with the pDA. Finally, the plates were washed several times with dH_2_O to remove unconjugated peptides. The prepared plates were stored in the dark in a desiccator to dry and used within 7 days of preparation.

### 2.5. Topography of Coating

The topography analysis of the pDA- and AMP-conjugated pDA coatings was performed with a Bruker Icon Atomic Force Microscope (Bruker UK Ltd., Coventry, UK). A silicone probe was passed over the surface of the coatings and its displacement was recorded. This generated a three-dimensional plot of the surface topography. In this research, images were recorded in static mode over a 20 μm × 20 μm area. Ellipsometry was performed on a Jobin-Yvon UVISEL ellipsometer (HORIBA UK Ltd., Northampton, UK) with a xenon light source. First, the light reflection of the uncoated surface of the sample was measured to set the measurement baseline, then the height of the coated surface was measured relative to the baseline.

### 2.6. Fluorescence Microscopy

Fluorescence microscopy was used to determine the conjugation of AMPs to the pDA with a Leica DM6000B widefield epifluorescence microscope (Leica Microsystems Ltd., Milton Keynes, UK), equipped with a 100 W short-arc epifluorescence mercury burner and a Leica DFL350 FX firewire camera (Leica Microsystems Ltd., Milton Keynes, UK) located at the Institute of Biomedical Research (IBR) in the School of Medicine, University of Birmingham. Measurements were performed with an epifluorescence filter set at an excitation wavelength of 480 nm and a green emission wavelength of 527 nm, corresponding to the green fluorescence associated with 5(6)-FAM. For each sample, a set of five random points were recorded for comparison and the brightness of the green light was analysed using the ImageJ 1.46r analysis program.

### 2.7. Scanning Electron Microscopy

Imaging of the uncoated and pDA-coated surfaces was performed on a Zeiss Supra 55VP scanning electron microscope (Carl Zeiss Ltd., Zeiss House, Cambridge, UK). All measurements were conducted on samples coated with ≈1 nm of the electrodeposited carbon film. A 10 kV electron beam and various magnifications were used.

### 2.8. Coating Stability 

In order to prepare the simulated body fluid (SBF), sodium chloride (NaCl), sodium bicarbonate (NaHCO_3_), potassium chloride (KCl), potassium phosphate dibasic trihydrate (K_2_HPO_4_·3H_2_O), magnesium chloride hexahydrate (MgCl_2_·6H_2_O), hydrochloric acid (HCl) 32%, calcium chloride (CaCl_2_), sodium sulfate (Na_2_SO_4_), and tris(hydroxymethyl)aminomethane (Tris, (CH_2_OH)_3_CNH_2_) were purchased from Sigma Aldrich, Merck Life Science UK Limited, Dorset, UK. The SBF was prepared as described by Kokubo and Takadama [20]. The stability of the AMPs on the surface of Ti6Al4V was performed in SBF by submerging and keeping AMP-coated plates in SBF at 37 °C for a total of 30 days. Analysis of the released AMPs to the solution was performed using two methodologies. Firstly, the change in green light intensity of the pDA Ti6Al4V surfaces coated with 5(6)-FAM-labelled peptides was analysed after 1 and 6 h, then at 1, 3, 7, 14, and 30 days of submersion in SBF, imaged under fluorescence microscopy. Secondly, plates with no labelled peptides were immersed in SBF solution and analysed by HPLC at the same time intervals. However, in this method, 100 μL of the SBF solution was withdrawn to determine the cumulative absorbance at the same retention time for every individual peptide.

### 2.9. Dynamic Contact Angle Analysis

Hydrophobicity was determined by a dynamic contact angle technique on a Dyne Technology ThetaLite optical tensiometer (Dyne Testing Ltd., Lichfield, UK) located in the Science City Lab at the School of Chemical Engineering, University of Birmingham. Deionised water was used as a liquid medium to perform the contact angle measurements. The measurements were performed by recording the contact and retraction angles of a 5 μL droplet released on and removed from the studied surface. The recording time of each incident was set to 20 s and the camera recorded 10 frames/s. The droplets were released and then removed at a speed of 5 mL/min. All surfaces were kept in a desiccator prior to measurements and were each measured three times.

### 2.10. Cell Culture Studies 

Human osteosarcoma cells (HOS) were purchased from the European Collection of Authenticated Cell Cultures (catalogue number 87070202). Cultured cells were grown in RPMI medium supplemented with 10% *v*/*v* fetal calf serum, 100 U/mL penicillin, 100 μg/mL streptomycin, and 2 mM glutamine as a monolayer in T75 cell culture flasks in a humidified atmosphere (5% CO_2_ incubator; 95% air) at 37 °C. Cells were sub-cultured at approximately 80% confluency twice-weekly using a standard trypsin-EDTA protocol. All cell cultures were confirmed as being free from Mycoplasma sp. contamination using the EZ-PCR mycoplasma detection kit according to the manufacturer’s instructions (Biological Industries USA, Cromwell, CT, USA). All cells were cultured up to passage 20 before being discarded. Before cell culture, coated surfaces were placed flat with the coated surface facing upwards into a 6-well cell culture dish. Surfaces were sterilised with 2 mL of 70% *v*/*v* ethanol for 30 min. Subsequently, surfaces were washed with sterile phosphate-buffered saline (PBS, 3 × 2 mL). HOS cells (100,000) were added and left to attach for 4 h. The medium was changed and cells were incubated for 5 and 7 days before being prepared for analysis by electron microscopy.

## 3. Results

### 3.1. Antimicrobial Characterisation

**Figure 1:**
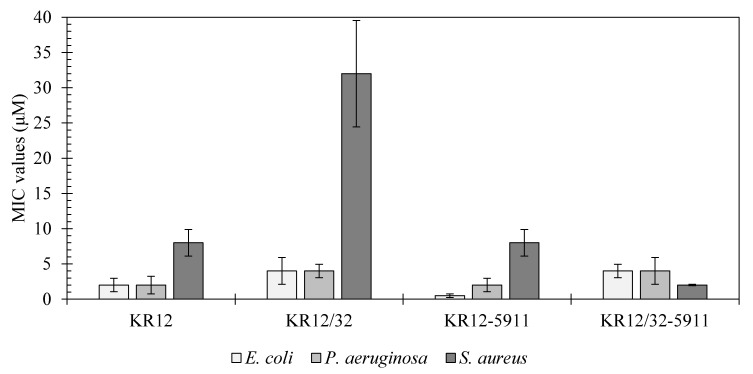
Minimum inhibitory concentration (MIC) values for the designed KR12 analogues.

The MIC values of the peptides KR12, KR12/32, KR12-5911, and KR12/32-5911 against *E. coli*, *P. aeruginosa,* and *S. aureus* are shown in Figure 1 and compared against LL-37 in Table 3. The most effective peptide against *E. coli* was peptide KR12-5911 at 0.5 μM, followed by KR12 at 2 μM, and KR12/32 and KR12/32-5911 at 4 μM. The lowest MIC values against *P. aeruginosa* were observed for peptides KR12 and KR12-5911 at 2 μM, whilst peptides KR12/32 and KR12/32-5911 showed MIC values of 4 μM. For *S. aureus,* the most effective was peptide KR12/32-5911 with an MIC of 2 μM, followed by KR12-5911 and KR12 at 8 μM and KR12/32 at 32 μM. All peptides show similar MIC values against Gram-negative bacteria. Additionally, the peptides were more effective against Gram-negative bacteria when compared to the Gram-positive bacteria, apart from KR12/32-5911. 

### 3.2. Polymerisation of Dopamine

**Figure 2:**
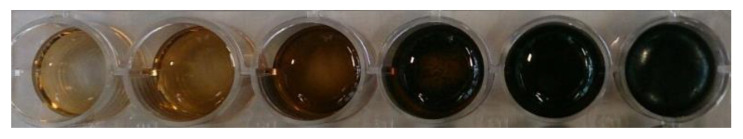
A photograph of s 24-well plate showing the change in colour of the alkaline dopamine solutions over 6 h, 12 h, 24 h, 48 h, 72 h, and 96 h, shown from left to right, respectively.

**Figure 3:**
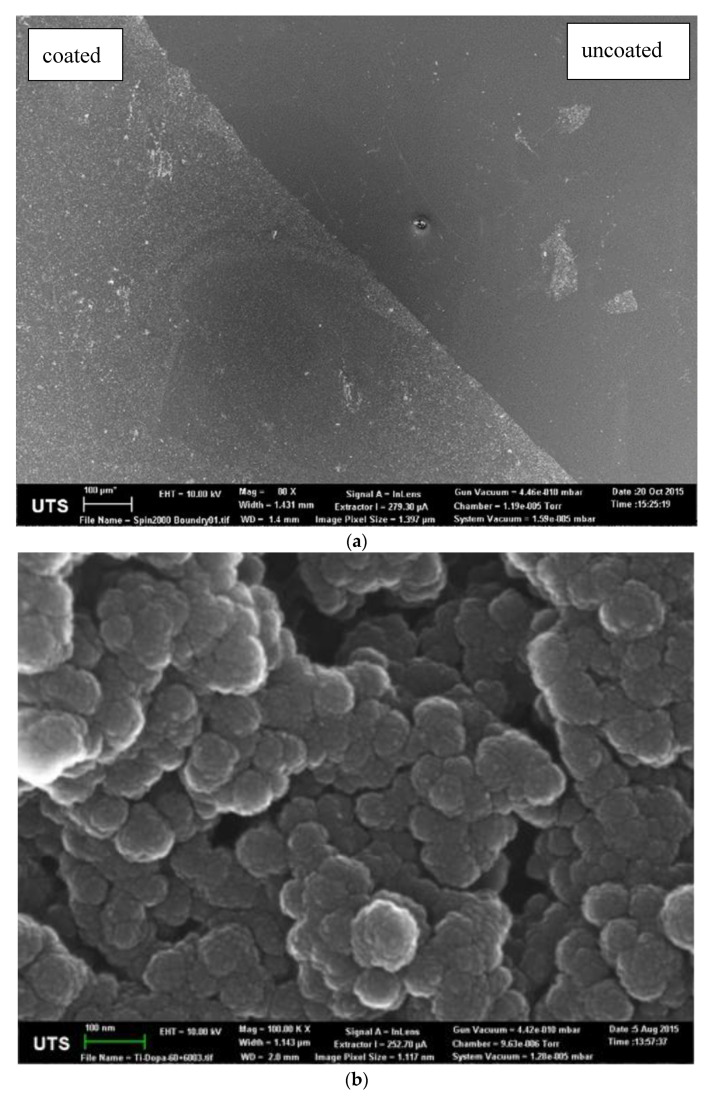
SEM micrographs showing (**a**) the Ti6Al4V surface with a polydopamine (pDA) coating (left) and uncoated (right) at a magnification of 1000×, as well as (**b**) the pDA coating at 100,000× magnification.

Dissolution of dopamine in the Tris buffer resulted in an immediate colour shift to light brown. As the polymerisation proceeded, the colour became darker until the solution was black, as shown in Figure 2. As can be observed, once the polymerisation took place for 96 h, a film formed between the surface of the liquid and the air, resulting in a different reflection of light. Scanning electron microscopy (SEM) micrographs of the pDA coatings are shown in Figure 3. Figure 3a shows a clear boundary between the uncoated and uniformly coated Ti6Al4V surfaces, while Figure 3b shows the bead-like structure of the polydopamine (pDA) coating. The beads display a circular shape with an average diameter of 86 ± 20 nm. It can be observed that the beads are in fact constructed of clusters of even smaller particles with an average diameter of 10 ± 1 nm. Even though the clusters are densely packed, there are visible inequalities in the shapes of the grooves in between the clusters. 

The thickness of the pDA coating was measured by both ellipsometry and AFM, the results of which are shown in Table 4. The results for the thickness of the coating measured by both ellipsometry and AFM agreed with one another. A 24-h immersion of Ti6Al4V plates in the dopamine solution resulted in the growth of an approximately 10-nm-thick layer. The thickness of the coating steadily increased to reach around 55 nm after 72 h, after which the growth plateaued.

### 3.3. Fluorescence Microscopy

**Figure 4:**
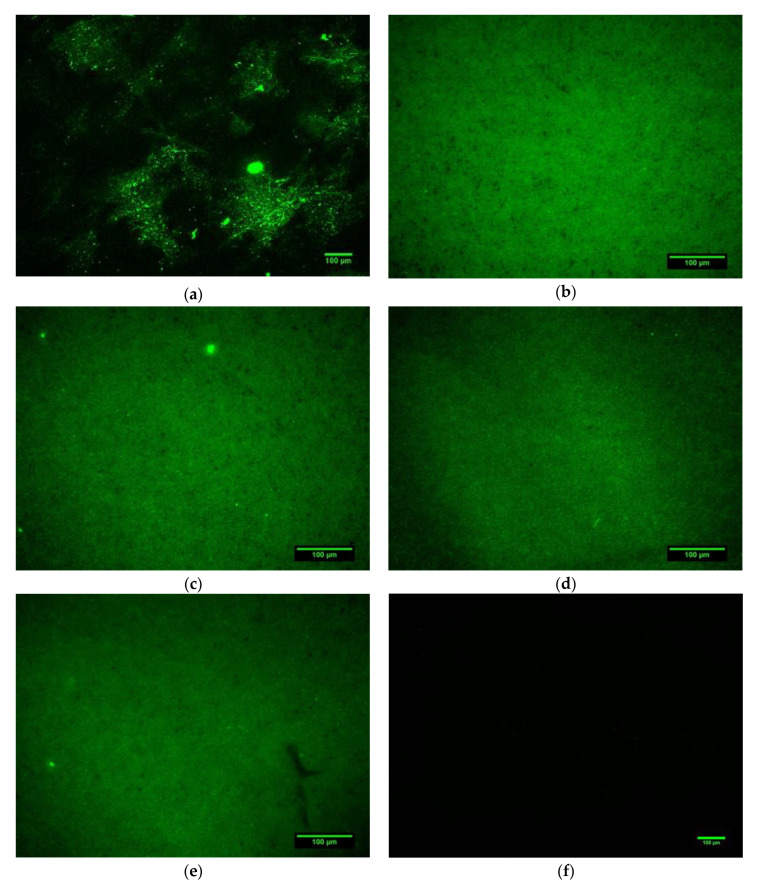
Fluorescence microscopy images of the Ti6Al4V surface coated for 24 h with 5(6)-FAM-labelled (**a**) KR12, (**b**) pDA-KR12, (**c**) pDA-KR12/32, (**d**) pDA-KR12-5911, and (**e**) pDA-KR12/32-5911; and (**f**) for Ti6Al4V without coating.

Fluorescence microscopy images of the various coated Ti6Al4V surfaces are shown in Figure 4a–f, while the subsequent fluorescence intensities are shown below in Table 5. From the fluorescence intensities of the peptide coating without the polydopamine, it can be observed that the peptide KR12 (Figure 4a) was present on the surface in the smallest quantities, while the lines presented in the image originated from the surface roughness and defects of the unpolished Ti6Al4V surface prior to coating. For the Ti6Al4V surfaces with a pDA linking substrate (Figure 4b–e), the green colour of the carboxyfluorescein (5(6)-FAM)-labelled peptides was generally distributed uniformly across the surface of Ti6Al4V, indicating a uniform peptide coating. 

### 3.4. Peptide Release Studies

**Figure 5:**
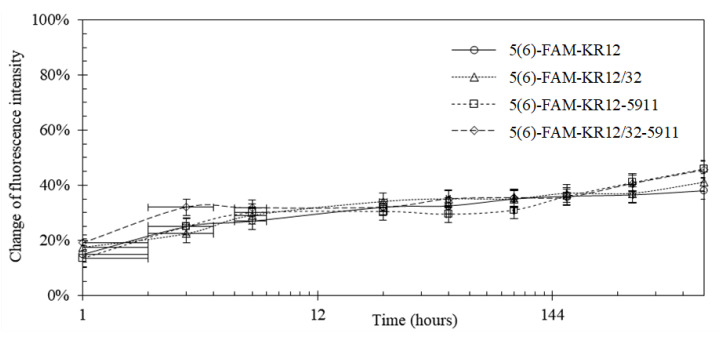
Release profile of 5(6)-FAM-labelled peptides that were released from the Ti6Al4V-coated surface based on fluorescence measurements; x-axis displayed in logarithmic scale (base = 12).

**Figure 6:**
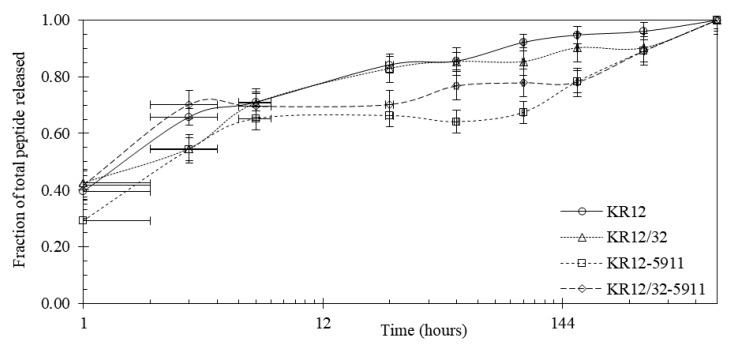
Release profile showing the percentage of the total peptides released in SBF from the pDA-coated Ti6Al4V surface based on HPLC; x-axis displayed in logarithmic scale (base = 12).

The peptide release profiles were all studied in simulated body fluid (SBF) over a total period of 30 days. The fluorescence-based release profiles of the pDA-attached peptides on the Ti6Al4V surface are shown in Figure 5. Roughly 30% of the peptides were released from the surface in the initial 6 h and 40–50% was released after 30 days. To verify the release profiles based on fluorescence, the studies were repeated with no labelled peptides and analysed by HPLC. The release profiles based on cumulative absorbance measured by HPLC are shown in Figure 6, which indicated that roughly 70% of the cumulative peptides were released in the first 6 h. 

### 3.5. Wettability of the Surface

**Figure 7:**
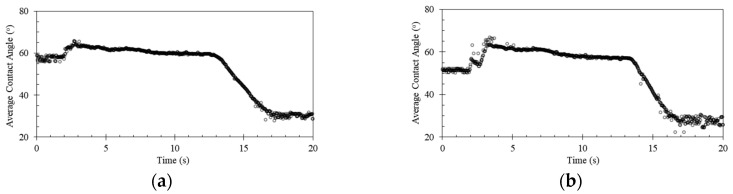

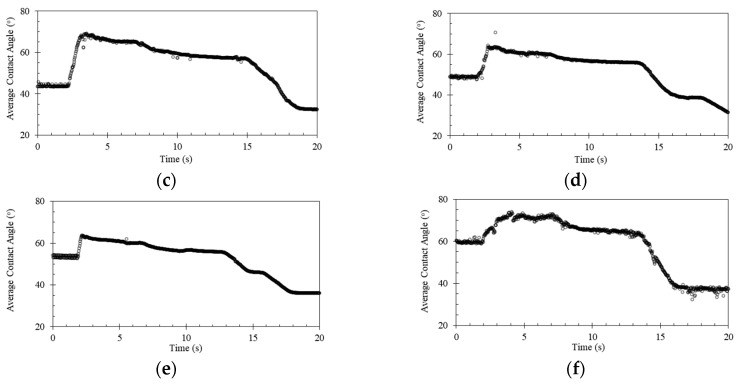
Changes of the water contact angles of the Ti6Al4V surface coated with (**a**) pDA, (**b**) pDA-KR12, (**c**) pDA-KR12/32, (**d**) pDA-KR12-5911, (**e**) pDA-KR12/32-5911, and (**f**) Ti6Al4V without coating, showing the changes in the water droplet’s volume.

The changes of the water droplet contact angles on the uncoated and coated Ti6Al4V with pDA and conjugated with KR12, KR12/32, KR12-5911, and KR12/32-5911 are shown in Figure 7. Three main events can be observed in the graphs: expansion of the droplet’s volume from 1 µL to 6 µL, the droplet’s volume of 6 µL remaining steady, and shrinkage of droplet’s volume from 6 µL to 1 µL. The application of the pDA and conjugation of the peptides all decreased the water contact angle of the surface of the Ti6Al4V, as shown in Table 6. Peptides KR12 and KR12/32 showed similar decreases in contact angles of roughly 1.3° and 7.8° when compared to pDA and Ti6Al4V, respectively. The coating containing peptide KR12/32-5911 had a contact angle about 1.0° smaller than those with peptides KR12 and KR12/32. However, the surface coated with the peptide KR12/32-5911 showed the biggest contact angle changes of roughly 2.7° and 9.7° when compared to the Ti6Al4V sample with pDA coating and the uncoated Ti6Al4V sample that was polished to mirror finish, respectively.

### 3.6. Cell Studies

**Figure 8:**
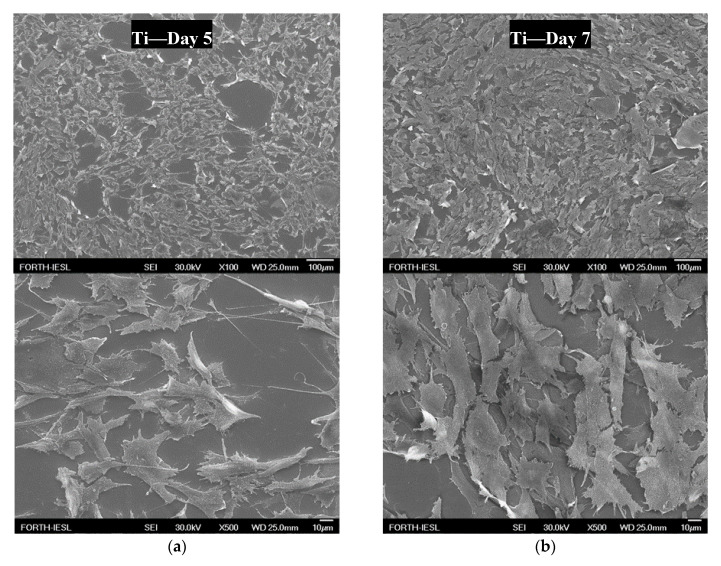

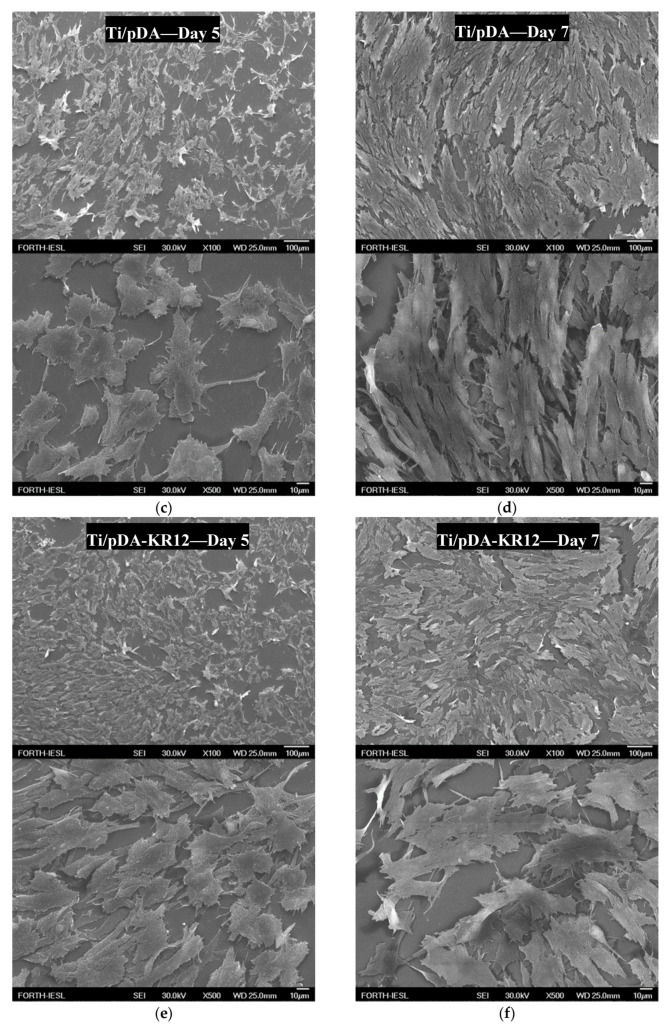

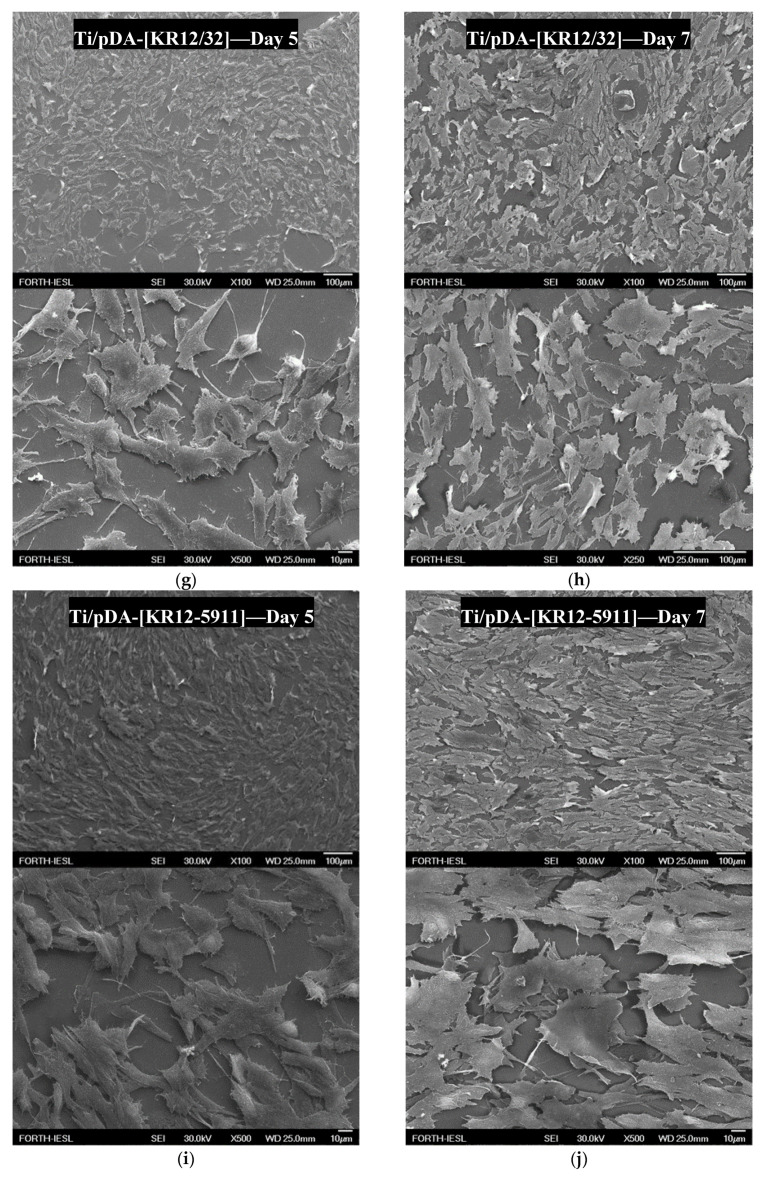

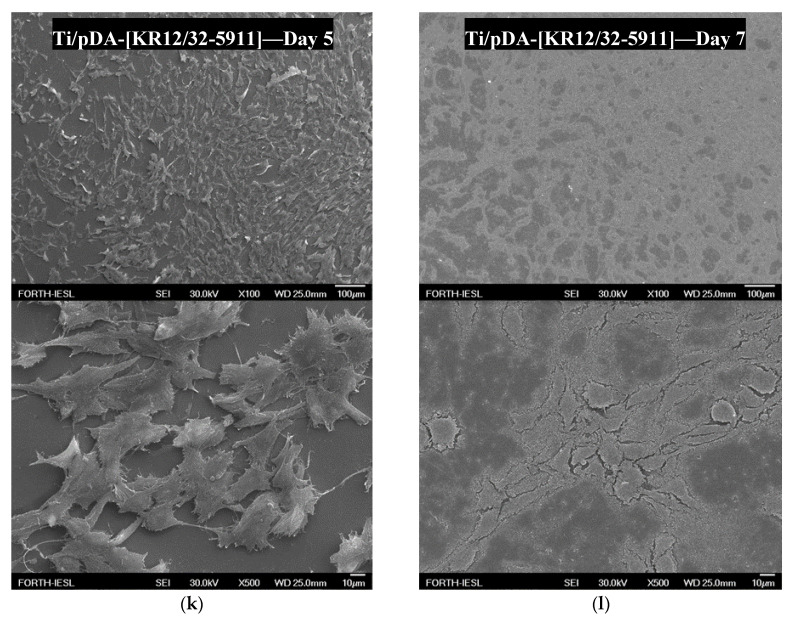
SEM micrographs showing the growth of the seeded human osteosarcoma cells (HOS) on day 5 and 7 for (**a**,**b**) Ti6Al4V, (**c**,**d**) Ti6Al4V/pDA, (**e**,**f**) Ti6Al4V/pDA-KR12, (**g**,**h**) Ti6Al4V/pDA-KR12/32, (**i**,**j**) Ti6Al4V/pDA-KR12/5911, and (**k**,**l**) Ti6Al4V/pDA-KR12/32-5911 at 100× and 500× magnification, respectively.

The micrographs presented in Figure 8a–l show the attachment of human osteosarcoma cells (HOS) on the coated and uncoated Ti6Al4V surfaces taken at days 5 and 7 of incubation. HOS showed consistent growth on all of the surfaces, except for the Ti/pDA surface decorated with the peptide KR12/32-5911 shown in Figure 8k–l, where cells were observed to be detached on day 7 of culture. On the Ti/pDA KR12/32 sample shown Figure 8g–h, the surface cells showed a flatter morphology compared to the other surfaces. On all of the other surfaces, although minor differences in the densities of cells can be seen, there is little change between the growth of cells and there is clear evidence of an increase in the number of cells from day 5 to day 7 of culture.

## 4. Discussion

### 4.1. Peptide MIC Characterisation

Infections caused by the investigated bacteria are some of the most commonly occurring incidences during orthopaedic joint replacement surgeries [21]. Factors such as prolonged antibiotic treatment or administration below the MIC value have been shown to increase bacterial resistance towards antibiotics [22]. Thus, it is important to design antimicrobial peptides with low MIC values that are effective against orthopaedic bacteria. When comparing the MIC values of the human cathelicidin LL-37 with the designed peptides, as summarised in Table 3, the KR12 peptide showed promising MIC values with a higher antimicrobial tendency towards Gram-negative bacteria. 

LL-37 was shown to have antimicrobial activity against fungi [23], bacteria [24], and viruses [23,25]. Additionally, it has immunostimulatory and immunomodulatory functions against infections [26] and stimulates angiogenesis [27] during the wound healing process [28]. LL-37 immobilised on polymer surfaces has also been proven to retain its antimicrobial activity [29]. Therefore, the designed KR12 analogous peptide may share similar properties with LL-37, but this remains to be investigated.

### 4.2. Polydopamine Coating of Ti6Al4V

Dissolving dopamine in a solution of alkaline pH changes the colour of the solution from translucent white to brown, then eventually to black, as shown in Figure 2. This colour change can be explained by the structure of dopamine. Dopamine contains phenol groups, and when polymerised it creates polyphenols structures that contain many aromatic rings joined together [30]. Multiple aromatic rings in close proximity in one molecule are known as charge transfer complexes, whereby the charge is delocalised between the rings [31]. These complexes promote absorption of visible light, causing electron transition from a lower electron state to higher electron state, as a result of which a dark colour is observed [32]. Immersing Ti6Al4V plates in a solution of dopamine resulted in the formation of a thin film on the surface of the metallic plate, which agrees with Messersmith et al. [33], who reported that pDA was able to adhere to virtually any surface and that its polymerisation in slightly basic solution resulted in a film formation on the surface. The SEM morphology and topology studies showed that the surface of the pDA coating was built from small round particles of ≈100 nm diameter. It was proposed by Jiang et al. that the pDA first polymerises in nanoaggregates, which over time attach themselves on the surface, creating a uniform coating [34,35]. 

The attachment of the nanoaggregates to the surface could potentially have been driven by the sedimentation of the particles towards the bottom of the container where the substrate to be coated was located. The coating then accumulated with more beads and increased the thickness of the coating, which varied depending on the size of the beads accumulated. This could explain the ≈10 nm coating thickness after the initial 24 h, and the increases of the thickness to 32 nm after 48 h and to 57 nm after 52 h of pDA polymerisation. Similar film thickness growth was reported by Jiang et al. [33,35]. In this study, a thickness plateau was reached after 72 h of polymerisation. This pattern of growth was also previously reported by Bensmann et al. [36], where the pDA film reached 62.8 nm in thickness after 72 h and did not increase in thickness afterwards. Formation of the plateau can be explained by the lack of oxygen after the formation of the first pDA layer. Since oxidation is a common reaction of polyphenols [37], it can be assumed that the polymerisation required oxygen to continue and the presence of the film limited the accessibility to oxygen. In addition, as oxidation on the surface will continue as oxygen is available on the surface, polymerisation will eventually stop when dopamine is consumed. 

### 4.3. Peptide Attachment to Polydopamine

The KR12 analogous peptide labelled with 5(6)-FAM at the N-terminal was conjugated to an already pDA-coated Ti6Al4V surface. The presence of the peptides was confirmed by the emitted fluorescence, showing only small variations in the measured fluorescence intensities between the different peptides. The quinone group of pDA is known to undergo a Schiff base reaction or Michael addition without the use of any other reagent or catalyst, allowing the conjugation of biomolecules to pDA [38]. The catechol group of the pDA is known to oxidise to quinone at a pH above 7.5 in excess of oxygen, which is necessary for successful conjugation to nucleophiles [39]. Here, the designed peptides contained two lysine residues that should be able to conjugate to pDA, but the first lysine was presumed to be shielded by the bulky 5(6)-FAM label at the N-terminus. Therefore, it was expected that pDA was conjugated with the second lysine residue relative to the N-terminus. It was observed that the initial burst of peptides released approximately 25–35% of the peptides from the surface after 6 h, and approximately 40–50% was released after 30 days. The fluorescence release profile studies in SBF indicated that 50–60% of the peptides potentially remained conjugated to the pDA after 30 days. The initial burst of peptide release could be attributed to the release of unattached peptides to the pDA that were not successfully removed during the wash step. After the initial burst, the rate of release of the peptides decreased and was only reduced further by roughly 5–10% after the final 30 days. The release profile from the fluorescence was confirmed by HPLC using non-fluorescent peptides. An initial ≈70% burst of cumulative peptide was released in the first 6 h, and a slower subsequent release for the remaining period was observed. These release studies clearly showed that these peptides were conjugated to the pDA-coated Ti6Al4V surface in a very stable manner through the lysine residues.

### 4.4. Wettability of the Surface

New implants within the body come into immediate contact with extracellular fluid and moieties, such as proteins, which are some of the first molecules to interact with the surfaces of an implant [40,41]. The optimal water contact angle of a biomaterial reported for bone-forming cells to attach to a surface was reported to be 55° [42]. Metallic surfaces are very hydrophobic, resulting in slow integration rates of the implant with the body. A water contact angle study of pDA-coated titanium surfaces by Nijhuis et al. reported that coated Ti6Al4V surfaces were much less hydrophobic (≈47°) than the uncoated surfaces (≈75°) [43], presumably resulting in an improvement of the implant integration [44]. In this paper, we report that the introduction of pDA coating on the surface of Ti6Al4V resulted in a decrease in the water contact angle from 65.4° ± 1.6 to 59.0° ± 1.2. The experimental values are in agreement with prior research performed by Luo et al. [45], who reported on the water contact angle of a pDA-coated Ti6Al4V surface. The water contact angle was further reduced to approximately 56.8° when the pDA coating was decorated with the peptides—the results varied depending on the used peptide, but they all showed a reduction relative to the pDA-coated Ti6Al4V surface. 

### 4.5. Cell Culture Studies

All surfaces showed attachment of cells on day 5, with no evidence of toxicity (which is determined by any change of cell morphology or density compared to the plain titanium surface), with one exception for the KR12/32-5911 surface. The cell morphology was generally "flattened" and fibroblast-like, typical of the described HOS morphology in the literature [46]. The density of attached cells increased over time, confirming that cells were viable and able to proliferate. In contrast, there was evidence that the surface of KR12/32-5911 was toxic to the cells, and at day 7 there were no longer any cells.

## 5. Conclusions

In the present research, KR12 and three new analogous peptides were successfully synthesised and showed promising antimicrobial activity against *E. coli*, *P. aeruginosa*, and *S. aureus*, with MIC values generally being lower than that of their native peptide, the human cathelicidin LL-37. The titanium surfaces were coated with pDA and characterised. Subsequently, the pDA-coated surfaces were decorated with KR12 and the other three designed analogous peptides. The pDA coating provided a long-term linking substrate for the peptides, which was confirmed by fluorescence microscopy and HPLC. Cultured HOS cells showed good attachment and cell growth on the material surfaces, with no visible toxicity towards the cells, except for the KR12/32-5911 peptide. This surface treatment shows potential to provide long-term antimicrobial activity on many metallic and organic material surfaces and could be used in biomedical materials and implants. 

## Figures and Tables

**Table 1:** Table showing the used peptides sequences, including their charge, hydrophobicity, and amphiphacity, determined by hydrophobic characteristics. Within the sequences, positively charged residues are marked in blue, the negatively charged in red, and highly hydrophobic sequences are underlined.

Peptide	Sequence	Length of Sequence	Net Charge	Charge Density	Mean Hydrophobicity [H]	Helical Hydrophobic
KR12	KRIVQRIKDFLR	12 aa	+4	0.33	0.193	0.782
KR12/32	KIRVQRIKDFLR	12 aa	+4	0.33	0.193	0.429
KR12-5911	KR IV R I K F R	9 aa	+5	0.56	0.178	0.395
KR12/32-5911	K I R V R I K F R	9 aa	+5	0.56	0.178	0.092

**Table 2:** Purity and mass analysis of peptides.

Peptide	Theoretical Molecular Weight (g/mol)	Molecular Weight M Measured by MS (g/mol)	Purity Calculated by HPLC (%)
KR12	1572	M = 1574 [M + 2H]^2+^ = 788 [M + Na + H]^2+^ = 799	95
5(6)-FAM-labelled KR12	1904	M = 1903 [M + 2H]^2+^ = 952	95
KR12/32	1572	M = 1572 [M + 2H]^2+^ = 787 [M + 3H]^3+^ = 525	99
5(6)-FAM-labelled KR12/32	1904	M = 1903 [M + H]^+^ = 1904 [M + 2H]^2+^ = 952	95
KR12-5911	1216	M = 1215 [M + 2H]^2+^ = 609	97
5(6)-FAM-labelled KR12-5911	1548	M = 1547 [M + H]^+^ = 1549 [M + 2H]^2+^ = 775	95
KR12/32-5911	1216	M = 1215 [M + 2H]^2+^ = 609	98
5(6)-FAM-labelled KR12/32-5911	1548	M = 1547 [M + 2H]^2+^ = 775	96
KR12	1572	M = 1574 [M + 2H]^2+^ = 788 [M + Na + H]^2+^ = 799	95
5(6)-FAM-labelled KR12	1904	M = 1903 [M + 2H]^2+^ = 952	95

**Table 3:** MIC values for the designed KR12 analogues compared to the LL-37 human cathelicidin.

Sequence	MIC Values (μM)			
	*E. coli* (I364)	*P. aeruginosa* (PAO1)	*S. aureus* (F77)	Geometric Mean
KR12	2	2	8	4.0
KR12/32	4	4	32	13.33
KR12-5911	0.5	2	8	3.5
KR12/32-5911	4	4	2	3.33
LL-37 [14]	8	8	4	6.67

**Table 4:** Polydopamine coating thickness as measured by ellipsometry and Atomic Force M.icroscopy (AFM).

Time of Immersion	24 h	48 h	72 h	96 h
Coating thickness measured by ellipsometry (nm)	10.2 ± 1.1	32.8 ± 1.1	52.4 ± 7.0	54.7 ± 6.4
Coating thickness measured by AFM (nm)	10.3 ± 0.5	34.1 ± 2.0	55.4 ± 9.0	57.1 ± 6.8

**Table 5:** Fluorescent intensity values of various Ti6Al4V peptide-coated surfaces over 24 h.

	5(6)-FAM-KR12	pDA-5(6)-FAM-KR12	pDA-5(6)-FAM-KR12/32	pDA-5(6)-FAM-KR12-5911	pDA-5(6)-FAM-KR12/32-5911
**Fluorescence intensity (FI)**	868 ± 144	1204 ± 37	1198 ± 44	1227 ± 42	1189 ± 52

**Table 6:** Overview of the water contact angles during the steady droplet volume.

Surface	Contact Angle at Steady Volume of the Droplet
Mirror polished Ti6Al4V	65.4° ± 1.6
Ti6Al4V coated with pDA	59.0° ± 1.2
Ti6Al4V coated with pDA and peptide KR12	57.7° ± 0.9
Ti6Al4V coated with pDA and peptide KR12/32	57.5° ± 0.7
Ti6Al4V coated with pDA and peptide KR12-5911	55.6° ± 1.1
Ti6Al4V coated with pDA and peptide KR12/32-5911	56.3° ± 0.5
